# Report of the Pennsylvania Hospital for the Insane, for the Year 1849

**Published:** 1850-04

**Authors:** 


*Report of the Pennsylvania Hospital for the Insane, for the year* 1849. By Thomas S. Kirkbride, M. D., Physician to the Institution. Published by order of the Board of Managers.

The report of Dr. Kirkbride represents this institution in a most flourishing condition. During the past year additional buildings have been erected, and increased accommodations and facilities for classification afforded, that place it in the front rank of similar institutions. It now contains sixteen distinct wards—eight for each sex—and ample room for 200 patients, and all the attendants whom it may be desirable to employ in their care. The whole number in the hospital during the year was 408, and the average number under treatment during the entire period was 210.

Notwithstanding the presence of cholera in our midst, and indeed to a fearful extent in an institution within sight of them, not a single case occurred in the Pennsylvania Hospital for the Insane, nor indeed a single case of acute sickness; a fact which speaks volumes for the admirable hygienic arrangements of the institution, and the unwearying vigilance of the medical officers. We have on a previous occasion, like the present, taken occasion to present to our readers a review of the past history of the insane department of the Pennsylvania Hospital; at no time did it ever exhibit a more prosperous condition or more extended means of alleviating the sufferings of this most interesting class of sufferers, than it does at this period under the fostering care of its able superintendent; and we wish that time and space were now allowed us again, to lay before our readers the valuable information in regard to buildings, warming, ventilation, and the various details of moral and other treatment contained in this report; but as this cannot be done, we cannot do better than refer them to the Report itself, with the assurance that they will be amply repaid in its perusal by seeing all that has been and is doing for the relief of the insane.

